# Model-based individual life-spanning documentation in visceral surgery: a proof of concept

**DOI:** 10.1007/s11548-024-03214-y

**Published:** 2024-06-17

**Authors:** Maximilian Berlet, Alissa Jell, Lars Wagner, Lukas Bernhard, Jonas Fuchtmann, Luca Wegener, Hubertus Feussner, Helmut Friess, Dirk Wilhelm

**Affiliations:** 1 TUM School of Medicine and Health, Klinikum rechts der Isar, Research Group MITI, Klinikum rechts der Isar, Technical University of Munich, Ismaninger Str. 22, 81675 Munich, Germany; 2 TUM School of Medicine and Health, Klinikum rechts der Isar, Department of Surgery, Klinikum rechts der Isar, Technical University of Munich, Ismaninger Str. 22, 81675 Munich, Germany

**Keywords:** Surgical documentation, Surgical load, Anatomical model, SDM-M, Surgical twin

## Abstract

**Introduction:**

Surgical documentation has many implications. However, its primary function is to transfer information about surgical procedures to other medical professionals. Thereby, written reports describing procedures in detail are the current standard, impeding comprehensive understanding of patient-individual life-spanning surgical course, especially if surgeries are performed at a timely distance and in diverse facilities. Therefore, we developed a novel model-based approach for documentation of visceral surgeries, denoted as 'Surgical Documentation Markup-Modeling' (SDM-M).

**Material and methods:**

For scientific evaluation, we developed a web-based prototype software allowing for creating hierarchical anatomical models that can be modified by individual surgery-related markup information. Thus, a patient's cumulated 'surgical load' can be displayed on a timeline deploying interactive anatomical 3D models. To evaluate the possible impact on daily clinical routine, we performed an evaluation study with 24 surgeons and advanced medical students, elaborating on simulated complex surgical cases, once with classic written reports and once with our prototypical SDM-M software.

**Results:**

Leveraging SDM-M in an experimental environment reduced the time needed for elaborating simulated complex surgical cases from 354 ± 85 s with the classic approach to 277 ± 128 s. (*p* = 0.00109) The perceived task load measured by the Raw NASA-TLX was reduced significantly (*p* = 0.00003) with decreased mental (*p* = 0.00004) and physical (*p* = 0.01403) demand. Also, time demand (*p* = 0.00041), performance (*p* = 0.00161), effort (*p* = 0.00024), and frustration (*p* = 0.00031) were improved significantly.

**Discussion:**

Model-based approaches for life-spanning surgical documentation could improve the daily clinical elaboration and understanding of complex cases in visceral surgery. Besides reduced workload and time sparing, even a more structured assessment of individual surgical cases could foster improved planning of further surgeries, information transfer, and even scientific evaluation, considering the cumulative 'surgical load.'

**Conclusion:**

Life-spanning model-based documentation of visceral surgical cases could significantly improve surgery and workload.

## Introduction

Surgical documentation has manifold implications, as it is the basis for judicial, reimbursement-related, and scientific assessment of surgical cases. [1] Nevertheless, its primary purpose is to transfer information about a particular surgery to other medical professionals, and surgical notes must be sufficient to comprehensively understand a procedure and the resulting postoperative anatomical situation. [2] If a patient has undergone many complex surgeries, fully understanding of the case can become tedious and time-consuming, as all relevant surgical reports must be available and read attentively. Surgical healthcare records from long-ago operations are often challenging to obtain if they still exist, making it difficult to illuminate an entire individual 'surgical life span.' [3] Furthermore, the current quasi-standard of narrative reports impedes quick answering of even elementary questions like the cumulative amount of organ resection or the exact position of anastomoses in visceral surgery, because the focus mainly lies on the procedure rather than the postoperative results. [4] Therefore, understanding modifications of a patient's anatomy during numerous surgeries requires reading each relevant surgical report from the first to the last line, as there is virtually no comprehensive representation. Moreover, managing classical and mostly paper-based documentation is of high effort, causes high economic costs, and complicates handover processes between medical professionals. [5] This all can lead to a critical lack of information, especially in emergency situations. Photographs or video clips have the potential to facilitate understanding of particular surgical procedures. However, no general standard exists to systematically attach them to surgical notes. A fundamental issue with this approach is the fact that recordings require commenting by the conducting surgeons and do not display the overall postoperative state. [6] Regarding the dimension of time, a further challenge is that all surgical notes must be ordered by date to sufficiently reproduce the accumulating 'surgical load' that patients sample during their life span. The concept of a cumulative 'load' with increasing morbidity after complex surgeries was already considered by former research on abdominal surgery. [7]

Even though modern technologies offer promising solutions, for example an electronic patient file that could store all surgical notes, this would only solve the availability problem but would not significantly improve the other issues mentioned. [8, 9] Therefore, complementing current procedure-oriented documentation with more result-focused solutions is mandatory. After an extended postoperative period, modifications to a patient’s anatomy might be more relevant than how the procedure was conducted in detail. In a word, the patient's cumulative surgical load must be accessible with one single view to allow for quickly answering exemplary questions like *'How long is the residual small intestine?'* after several ileum segment resections, or *'How far away from the ligament of Treitz is a Roux-en-Y anastomosis located?'* after a total gastrectomy.

To approach this problem of concise yet comprehensive life-spanning documentation, we developed a functional prototypic software for research deploying a model-based documentation concept building upon a basic anatomy (BA) with derived interactive 3D computer models of an individual patient's pre and postoperative states. We named our approach 'Surgical Documentation Markup-Modeling' (SDM-M), as it can easily be modified and expanded by patient-individual parameters stored in an XML-like file. [10] As a proof of concept and to evaluate the possible clinical impact of such an approach, we conducted a study with actual surgeons and advanced medical students from our local Department of Surgery at the University Hospital Rechts der Isar of the Technical University of Munich, comparing classical paper-based narrative surgical notes with SDM-M, utilizing simulated complex surgical cases.

## Material and methods

### Development of an anatomy-based surgical documentation markup model

**Fig. 1:**
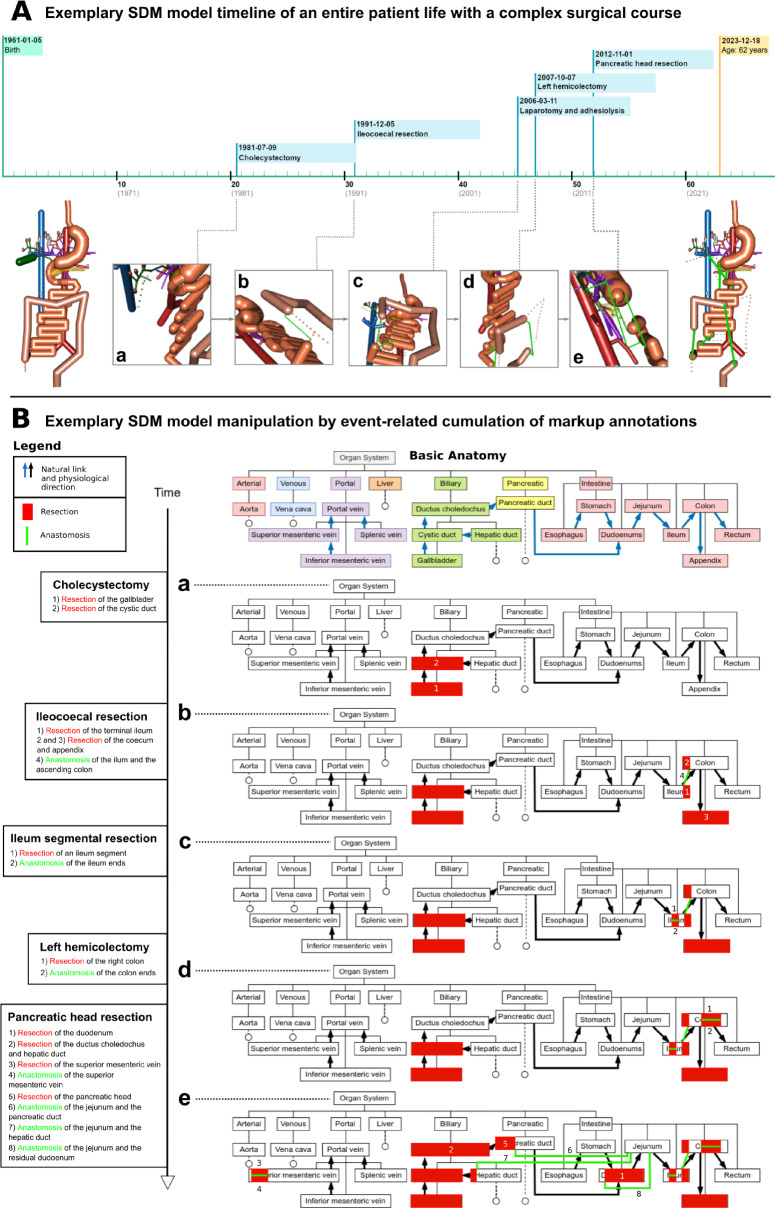
**A** Exemplary SDM model timeline of a patient with complex surgical course, **a**–**e** Interactive 3D models of the distinct postoperative anatomy after every single surgery, the patient underwent during their life. The full surgical report and the 3D models can be opened by clicking on the respective flag along the timeline. **B** Exemplary SDM model manipulation of a basic anatomy (BA) – Each surgery **a-e** increases the cumulative surgical load of the patient. When sharing the model with other medical professionals or facilities, only the colored information in the charts must be transferred. The example shows the lifetime of the simulated patient ‘P2’ from the evaluation study

We developed our software prototype for research from scratch using several programming languages and concepts. The developing process involved medical experts and engineers from our local Department of Surgery. Demands, regarding a possible future model-based surgical documentation platform, were thus directly derived from our local experience in complex visceral surgery and surgical documentation. The entire developing process from planning to a functional proof-of-concept prototype took 12 months. The core system for storing and administering the simulated patient models was developed in an 'apache' version 2.4.52 web server environment running on a virtual machine with Ubuntu GNU/Linux server edition 22.04. A configuration with 4 Gigabyte RAM and 1 CPU Core (AMD® Ryzen 7 3700u with a clock rate of 3.2 GHz) was enough to run the application stable and reliably. 'PHP' version 8.2.13 and a 'MYSQL' database system version 8.0.35 were deployed as the application's backbone on the server side. 3D rendering of the pre- and postoperative anatomical patient states was performed using a self-written R script (R version 4.1.2) with the additional packages 'rgl' version 1.2.1 (a visualization framework using OpenGL) and 'htmlwidgets' version 8.0.35. [11–13] The 3D models are calculated and displayed immediately after inserting modifications via the SDM-M web interface. The exclusive use of the Hypertext Markup Language (HTML) and JavaScript on the client side achieved compatibility with all modern web browsers, allowing for using the application on any computer or handheld device. The software prototype for research comprises three core functionalities:

*The first* is a database system to store and edit basic anatomies (BA) upon which individual patient models can be built. Anatomical structures are integrated into a hierarchical tree topology of organ systems. For our proof of concept, we included seven functional organ classes relevant to surgery on the digestive tract: 'Arterial,' 'Venous,' 'Portal,' 'Liver,' 'Biliary,' 'Pancreatic,' and 'Intestine.' The hierarchical concept represents proven anatomical relations known from medical standard books. To simulate the anatomical structures' functional (physiological) properties, they are connected via 'natural links' similarly to 'hyperlinks' in the World Wide Web. A link leads from one structure to another in the direction of their functional conjunction. For instance, a link from an artery *A* to another artery *B* represents the physiological blood flow in that direction. Accordingly, a natural link from the ileum to the ascending colon represents the physiological direction of stool propulsion. (Fig. 1B) This concept allows for functional reasoning and the creation of even comprehensive physiological models. The anatomical hierarchy and the natural links can easily be maintained by editing parent or child structures and entering spatial information like diameters and lengths.

*The second core function* is a 3D rendering system deploying OpenGL, which creates the output directly from the database using the BA and natural links. [12] An anatomic structure thereby ’originates’ from another it is connected to by a ’natural link. Dotted lines for resections and bold green lines for reconstructions illustrate patient-individual modifications of the BA during surgical procedures.

The 3D output is seamlessly integrated into the *third core functionality*, which is a comprehensive solution for writing and displaying surgical notes with attached 3D annotations for a deliberate number of patients. Reports can be authored using text input for each surgical step and a special input form to annotate resections of organ parts or insert 'artificial links' in terms of reconstruction. The modifications are immediately integrated into the patient-individual anatomical model and displayed in the graphical user interface (GUI). Each simulated patient has their own 'surgical timeline' with all surgeries available in the database being pointed out as timestamp flags in the correct order. The timeline is divided into decades, allowing for a straightforward reading of a patient's age at a particular surgery. On the left side, the user sees the anatomical state of the simulated patient at birth (without any alterations of the BA), while on the right side, the current state with all resections and reconstructions in terms of the cumulated surgical load is displayed. All 3D models can be moved and turned around interactively in a 3D environment using the PC’s mouse, a track pad, or the touch display of a handheld device. The user can easily zoom into the model to investigate even small structures and alterations. (Fig. 1A) Figure 1 shows the surgical life span of one simulated patient (P2), employed in the evaluation study which is presented below. By clicking on a surgery flag along the timeline, the user can open the particular surgical report with interactive 3D models of the anatomical state before and after this surgery. The panels **A/a** to **e** in Fig. 1 show the state after each simulated surgery, accessible by clicking on the respective flag. Figure 1B shows the modeling approach behind the scenes. Based on the standard BA, each surgery causes patient-individual modifications that cumulate with each further surgery in terms of the already mentioned surgical load. Only the colorful highlighted markup information in the panels **B/a** to **e** have to be transferred to allow for model reconstruction after transfer to another medical facility. Besides surgical information about resection and reconstruction, even natural anatomical biometrics, such as the length of bowel sections, or arterial supply of organs can be annotated and used to modify the BA individually.

### Design of the evaluation study

Two primary aspects must be considered to estimate a future possible impact of a life-spanning SDM-M for surgical documentation on daily clinical workflow. On the one hand, the data has to be inserted into the model, which is, at the moment, achieved by manual annotation under every single surgical step of a classic surgical report typed or pasted into an online interface. This approach requires little effort, but should be investigated more deeply in future research, especially regarding the aspect of automated data integration.

More important in the current state is the possible improvement in information transfer of a patient's surgical course to a medical professional who did not participate in the underlying surgeries. Thus, our research question was how our approach represented by the proposed software-prototype could improve the perception and understanding of the life-spanning surgical load of individual patients. Therefore, we designed an evaluation study at our local Department of Surgery at the University Hospital Klinikum rechts der Isar in Munich.

**Fig. 2:**
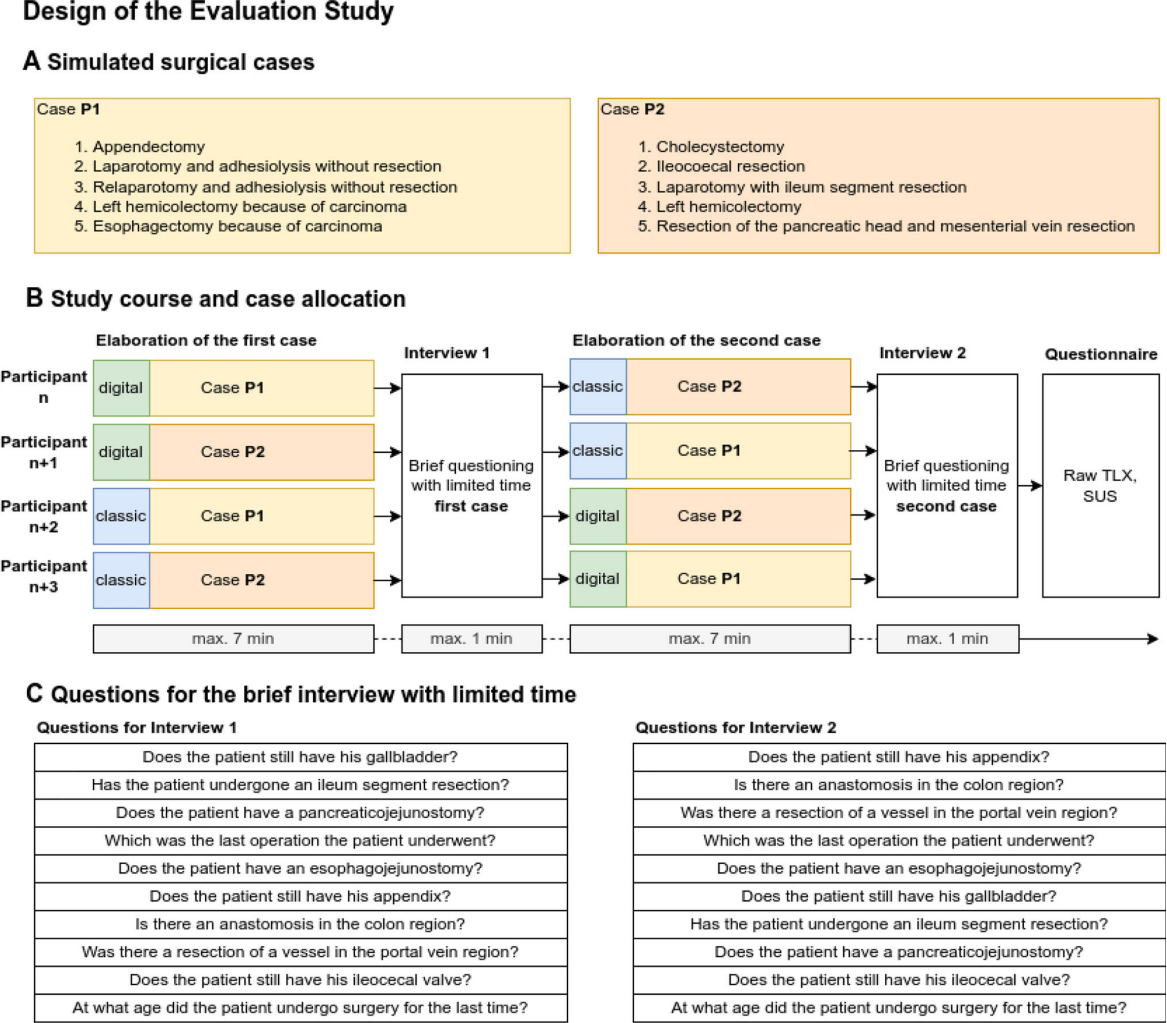
Design of the evaluation study – **A** Surgical course of the two simulated patients P1 and P2. The surgeries are listed in the timely order of their occurrence. **B** Study course and case allocation. Every participant (surgeon or advanced medical student) worked out both cases. The order of the approaches was thereby switched to exclude confounding by habituation. **C** For both turns, the same questions were posed, however, in a different order

We simulated two patient cases (P1 and P2) with similar complex but diverse surgical life-spanning courses. Both patients underwent five devised visceral surgeries with distinct modifications of the BA. (Fig. 2A) P1 underwent appendectomy, twice relaparotomy with adhesiolysis but without bowel resection, left hemicolectomy, and finally, an esophageal resection with gastric sleeve and gastroesophagostomy. P2 underwent cholecystectomy, ileocecal resection, relaparotomy with ileum segment resection, a left hemicolectomy for colon carcinoma, and a pancreatic head resection for cancer with partial resection of the superior mesenteric vein. The simulated surgeries were worked out once in a classic way using narrative surgical notes and once using our prototype software with anatomical annotations. The classic narrative surgical notes for P1 comprised 2700 words, and for P2, 2600 words. Participants for the evaluation study were surgeons and medical students in their last year before graduation from the local Department of Surgery. All participants are involved and experienced in the daily treatment of complex surgical cases. The participants were asked to elaborate and understand the two simulated cases and answer predefined questions in a personal interview with limited time. From an analysis of 2135 physician–patient contacts in our local outpatient department, we derived an average conceded time of 7 min to understand a case roughly at the first contact between patient and physician. Hence, the participants had a maximum of 7 min (420 s) to work out each case but could stop the process at any earlier time as it was comfortable for them. The cases and the approaches (classical paper-based vs. SDM-M) were switched among the subjects to reduce habituation and other confounding factors. Each participant worked out both cases (P1 and P2), one in the classic way and one digitally. (Fig. 2B) The predefined questions of the interview were always the same, but sorted in a different order for the first and second turn of the study. (Fig. 2C) After working out both cases within a maximum of 7 min and answering the standardized questions within 1 min, the participants filled out a questionnaire based on the Raw NASA-TLX (NASA Task-load Index) and the SUS (System Usability Scale). [14–16] Moreover, the participants commented on their daily use of modern technical devices like smartphones, tablets, PCs/laptops, and video conferencing software in their private and professional contexts. All participants signed informed consent before their inclusion in the proof-of-concept study. We deployed the statistical software R version 4.1.2 to evaluate the results. [11] Testing for statistical significance was performed using the paired Wilcoxon rank sum test, presuming a level of significance of 5%. The results are given as mean with standard deviation (± SD) or median with interquartile ranges (IQR). For the assessment of the internal consistency of the Raw NASA-TLX, Cronbach's alpha was calculated using the R package 'psych' version 2.3.9. [17, 18]

## Results

For the proof of concept, 24 participants were included in the evaluation study, of which 16 were physicians from the local surgical department and 8 were medical students in their last year before graduation. Table 1 depicts the complete baseline characteristics. All participants were experienced in the use of modern information technologies. Hundred percent stated they would use a personal computer or laptop privately and under professional circumstances. However, at least half of the participants did not use smart or handheld devices under professional circumstances.

**Table 1:** Baseline characteristics of the evaluation study

Parameter	Total	Physicians	Students
Number of participants		*n*	
	24	16	8
Age		%	
20–29 y	66.67	56.25	87.5
30–39 y	25	31.25	12.5
40–49 y	4.17	6.25	0
> 50 y	4.17	6.25	0
Professional experience in medicine		%	
< 2 y		56.25	
2–4 y		31.25	
5–6 y		6.25	
> 6y		6.25	
Private media use		%	
Smartphone	100	100	100
Tablet	45.83	37.5	62.5
PC\laptop	100	100	100
Video conferencing software	50	50	50
Professional media use		%	
Smartphone	33.33	37.5	25
Tablet	16.67	12.5	25
PC\laptop	100	100	100
Video conferencing software	50	50	50

The time required for elaborating and understanding the simulated surgical cases varied significantly between the classic paper-based and the SDM-M approach. While the average time needed with the classic method was 354 ± 85 s, it was reduced to 277 ± 128 s with the prototypic SDM-M software. (*p* = 0.00109) Of note, 11 participants exploited the inherent time using classic paper reports, while only 8 needed the full 7 min with the SDM-M software. Particularly, students required the complete time. (75% with the classic approach and 62.5% with SDM-M) An observed reduction in the error rate from 2 ± 2 to 1 ± 1 errors out of 10 interview questions using SDM-M was statistically insignificant. (*p* = 0.05325).

**Fig. 3:**
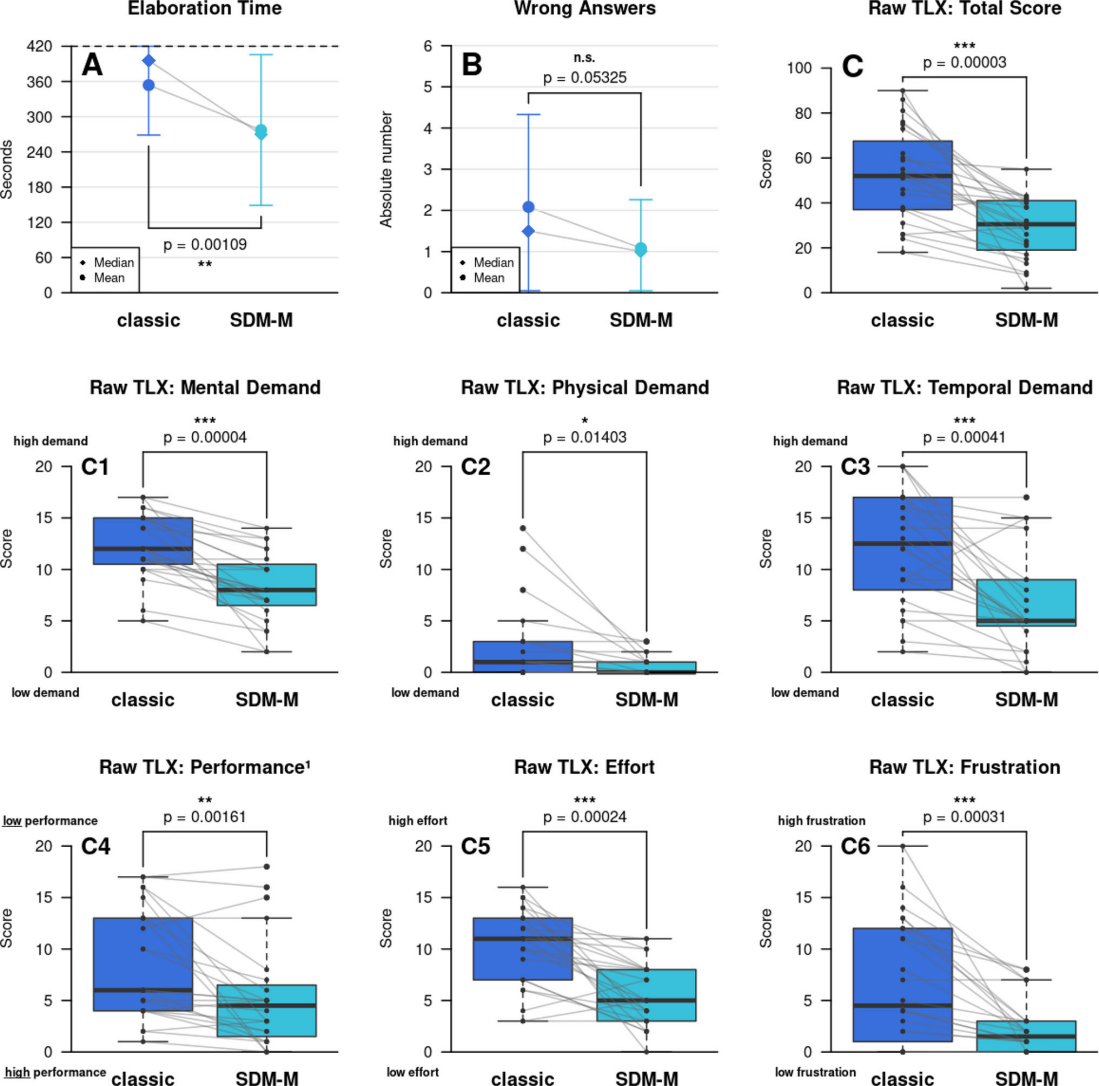
Results of the evaluation study – **A**–**B** the error bars indicate the standard deviation. **C**–**C6** The bold line in the middle of the box represents the median and the box itself the interquartile range. SDM-M = Surgical Documentation Markup-Modeling, * *p*-value < 0.05, ** *p*-value < 0.01, *** *p*-value < 0.001, Level of significance: 5%

The analysis of the perceived workload revealed a significant reduction in the overall Raw TLX score from a median of 52 (IQR 27.75) to 30.5 (IQR 21) when using SDM-M. (*p* = 0.00003) The mental demand decreased from a median of 12 (IQR 4.25) to 8 (IQR 3.5). (*p* = 0.00004) Even the physical demand was perceived to be lower with SDM-M, with a median score of 1 (IQR 3) for the classic and 0 (IQR 1) for the digital approach. (*p* = 0.01403) In accordance with the measured reduction in time demand, the perceived time pressure decreased with SDM-M, with a median of 12.5 (IQR 8.5) for the classical and 5 (IQR 4.25) for the novel approach. (*p* = 0.00041) Also, the perceived overall performance was improved by the model-based prototype, with a significant reduction from a median of 6 (IQR 9) to 4.5 (IQR 4.5). (*p* = 0.00161) Of note, in the NASA-TLX, a reduction in the performance score indicates an improvement. Finally, the perceived overall effort decreased from a median score of 11 (IQR 6) to 5 (IQR 5) (*p* = 0.00024), and the frustration during the elaboration and the interview with time pressure was significantly diminished, from a median of 4.5 (IQR 10.5) to 1.5 (IQR 3). (*p* = 0.00031) (Fig. 3) Cronbach's alpha for the Raw TLX was 0.79, indicating 'good' internal consistency. [17] Table 2 compares physicians and students regarding the impact of SDM-M on time-sparing, error-improvement, and the Raw TLX. Here, the two groups of medical professionals had no significant difference regarding the overall performance improvement, perception of time, error rate, or task load.

**Table 2:** Difference between physicians and medical students in the SDM-M evaluation study

Parameter	Physicians				Students			Group difference
	Dimension	Mean change	SD	*P* value	Mean change	SD	*P* value	*P* value
Time	Seconds	− 168.06	114.34	**0.00317**	− 31.25	69.08	0.18145	0.18145
Error	Number	− 1.25	1.06	0.17472	− 0.62	1.14	0.3222	0.75176
Total raw TLX	Score	− 27.44	25.48	**0.00058**	− 18	17.85	**0.02489**	0.37425
Mental demand	Score	− 5.62	4.65	**0.0007**	− 3.5	2.33	**0.02154**	0.70929
Physical demand	Score	− 0.12	1.31	**0.01402**	0	0	–	0.14221
Temporal demand	Score	− 6.5	6.6	**0.00109**	− 3.38	5.45	0.14221	0.3557
Performance	Score	− 4.19	8.26	**0.02399**	− 4.12	4.73	**0.03552**	0.53428
Effort	Score	− 6.81	3.85	**0.00372**	− 3.62	2.77	**0.02178**	0.78188
Frustration	Score	− 4.19	6.65	**0.00163**	− 3.38	4.21	0.09751	0.36729

Level of significance: 5%

**Fig. 4:**
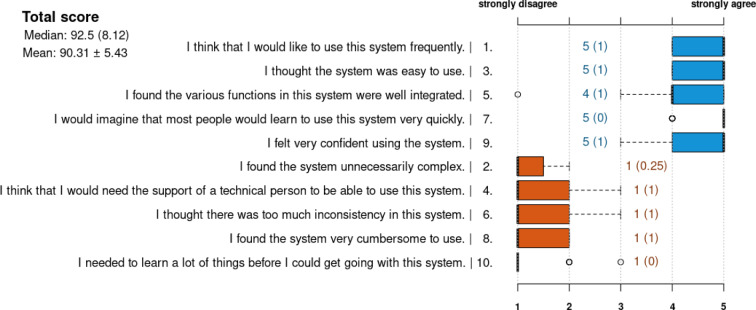
Result of the System Usability Scale after the evaluation study—The numbers in the plot represent medians with interquartile ranges in brackets. For better legibility, the original order of the questions was changed in the plot to separate positive (blue) from negative (orange) questions

According to the System Usability Scale (SUS), the participants indicated they would like to use a system like the proposed prototype frequently (Median 5 IQR 1) and felt confident in its application (Median 5 IQR 1). The total System Usability Score was at a median of 92.5 (IQR 8.12). (Fig. 4) In addition to the structured questionnaire, the participants were also encouraged to comment on possible improvements to the proposed software prototype. Accordingly, more information should be displayed in the life-spanning timeline, such as the particular indication for surgery or radiological image data. Also, other treatments and events were suggested as useful to be included in the model, like non-surgical interventions or emergencies. More expressive coloring was demanded regarding the annotation of resected organ parts and their reconstruction, and in the final cumulative model, a different coloring for every surgical step was considered beneficial by the participants.

## Discussion

In this article, we report a proof of concept for a Surgical Documentation Markup-Modeling approach (SDM-M) representing the surgical course of a patient's entire life span. To evaluate the possible impact on the daily clinical work of physicians obligated with surgical patients, we performed a study with 24 physicians and advanced medical students from our University Hospital's Department of Surgery.

The significant reduction in the time and effort required for assessing complex surgical cases by SDM-M indicates that a model-based approach could positively impact the increased workload in surgical departments. [19] Our study results revealed that a model-based approach is not inferior to the classic one in understanding a complex visceral surgical case while significantly reducing the perceived workload and frustration. Therefore, our software prototype allowed for a natural assessment to the simulated cases. Interestingly, the first step of elaborating cases with the classic paper-based approach during the study was sorting the narrative reports by date in most cases. Thus, our approach comprising life-spanning ordered timelines matched the medical professionals' comprehension of surgical case assessment. The time modeling component is crucial for sufficiently reproducing the cumulative surgical load and is an elementary component of a comprehensive result-oriented documentation. Thus, SDM-M could exceed the frontiers of merely procedural description and could expand the functionality of surgical documentation beyond the scope of current narrative reports. It links former independent surgical notes to a comprehensive 'pragmatic surgical twin' of an individual patient with a temporal context. [20]

Even the prototype’s system usability evaluation results promise a possible future clinical application of similar models. The current prototype was perceived to be intuitive and easy to use. However, the study participants emphasized several recommendations for improvement regarding the information presentation and density; our proof of concept was already considered functional. In our study, we only displayed organs relevant to the study cases in the field of visceral surgery. Representation of more and even interdisciplinary surgical interventions would require a more sophisticated rendering concept regarding concentration of information and interoperability. [21] A comprehensive surgical twin with surgeries on different body parts like the musculoskeletal or the nervous system could cause interference and confusion if 3D models are presented unwisely with a too high degree of detail. [22] Therefore, filter functions might be beneficial to only present information relevant to answer distinct questions. Another challenge will be finding a balance between procedural and result-oriented surgical documentation approaches. Therefore, a clever combination of both approaches might be most beneficial as procedural descriptions of particular surgeries could be used to calculate the resulting anatomical modifications and insert them directly into the life-spanning SDM-M. [23] The current research prototype is limited to a manual input of the procedure-related modifications via a web interface; however, in the current state, no programming capabilities are necessary to perform this on the user's side. Even entire anatomical structures can be easily added by creating new instances using our web GUI. More sophisticated applications could do this by modifying the BA over an API in the future. In this sense, even fully automated sensor-based or artificially intelligent applications could transfer the surgical load acquired during a surgery directly into the model. [24, 25]

Building upon our approach could also enable future surgeons to plan procedures based on the individual surgical load. Thus, the extent of bowel resection during surgery can be adapted to the expected residual length of the small intestine after multiple former segmental resections to prevent a short bowel syndrome, for instance. In this sense, our evaluation study reflected the actual daily routine quite well, as the questions asked during the timely limited interviews were all relevant when planning abdominal surgery. [26]

Inevitably, our study has some limitations. Thus, the model was so far only tested with visceral surgeons and students, currently obligated with cases from this field. Trauma or neurosurgery was not yet considered, leading to the necessity to evaluate similar models even for this purpose. Particularly in the case of soft tissue surgery, where spatial relations are problematic to determine due to a certain flexibility of the involved structures, the concept of 3D modeling on a basic anatomy could be more beneficial than in neurosurgery, where spatial relations are much more precise to determine. [27] Here, radiology-based approaches that are already in use might be more sufficient. However, we intended to primarily enable a distinct and structured approach for surgical documentation, transporting the 'surgical concept' underlying a procedure. [28] Hence, in future developments, even a detailed radiological biometry integration could lift the approach to the next level. [29]

All these findings stipulate the future tasks regarding the SDM-M approach. From the technical point of view, it is mandatory to even take care for security matters and a seamless integration into existing clinical software environments if planning to implement a productive model-based surgical documentation system. At the current stage, our prototype is only used as a proof of concept and merely simulated patient data were used for the models. However, to enable interoperability between different healthcare facilities, common standards must be defined to make such a system applicable and to guarantee performance and patients’ privacy. Therefore, all relevant data could be merged into one encrypted standardized file format, fostering exchange of comprehensive medical models. Especially regarding the rapidly proceeding digitalization in medicine, definition of such standards, similar to DICOM, is mandatory even for still unstructured concepts. [30] There already exist markup languages for medicine and surgery, but they are more tailored for education and training rather than for documentation and do not consider a life-spanning time component. [31] A further challenge would be to transfer current language-based documentation automatically to SDM-M, for example, via natural-language recognition. [32] Moreover, physiological models must be designed and evaluated to predict the possible impact of a planned surgery on the patient's morbidity. [33] Thus, for example, the simulation of peristaltic waves in a patient-individual model could predict the amount of biliary reflux after a planned gastric resection with Roux-en-Y reconstruction. Such modeling would also improve the comparability between individual surgical cases as a more operational scientific approach becomes possible. After all, our proof of concept forecasts a promising valuable impact of model-based documentation approaches and suggests that further developing of SDM-M could significantly contribute to the field of surgery.

## Conclusion

Our proposed life-spanning model approach for surgical documentation could improve the elaboration process and understanding of complex abdominal surgeries with resection and reconstruction on a patient-individual timeline. Our research software prototype significantly reduced effort and perceived task load of medical professionals in an evaluation study. Thus, model-based approaches promise to improve future individualized surgical care and inter-facility exchange of surgical information.
